# Diagnostic value of interferon-*γ* release assay in HIV-infected individuals complicated with active tuberculosis: a systematic review and meta-analysis

**DOI:** 10.1017/S0950268821001953

**Published:** 2021-08-23

**Authors:** Yiyun Ma, Yuni Xu, Xiaoqiang Cao, Xiaojuan Chen, Yeteng Zhong

**Affiliations:** 1 Department of Laboratory Medicine, Hainan General Hospital, Haikou, Hainan, China; 2 Department of Laboratory Medicine, The Second Affiliated Hospital of Hainan Medical University, Haikou, Hainan, China

**Keywords:** Human immunodeficiency virus, interferon-*γ* release assay, meta-analysis, tuberculosis

## Abstract

**Objective:**

Although the interferon-*γ* release assay (IGRA) has become a common diagnostic method for tuberculosis, its value in the diagnosis of tuberculosis in human immunodeficiency virus (HIV) seropositive patients remains controversial. Therefore, this systematically reviews the data for exploring the diagnostic value of IGRA in HIV-infected individuals complicated with active tuberculosis, aiming to provide a clinical basis for future clinical diagnosis of the disease.

**Methods:**

Relevant studies on IGRA for diagnosing tuberculosis in HIV-infected patients were comprehensively collected from Excerpta Medica Database (EMBASE), Medline, Cochrane Library, Chinese Sci-tech Periodical Full-text Database, Chinese Periodical Full-text Database, China National Knowledge Infrastructure (CNKI) and China Wanfang Data up to July 2020. Subsequently, Stata 15.0, an integrated statistical software, was used to analyse the sensitivity, specificity, diagnostic odds ratio (DOR), positive likelihood ratio (PLR) and negative likelihood ratio (NLR) to create receiver operator characteristic (ROC) curves.

**Results:**

A total of 18 high-quality articles were selected, including 20 studies, 11 of which were related to QuantiFERON-TB Gold In-Tube (QFT-GIT) and nine to T-SPOT.TB. The meta-analysis indicated that the pooled sensitivity = 0.75 (95% CI 0.63–0.85), the pooled specificity = 0.82 (95% CI 0.66–0.92), PLR = 4.25 (95% CI 1.97–9.18), NLR = 0.30 (95% CI 0.18–0.50), DOR = 14.21 (95% CI 4.38–46.09) and the area under summary ROC curve was 0.85 (95% CI 0.81–0.88).

**Conclusion:**

IGRA has a good diagnostic value and therefore can aid in the preliminary screening of active tuberculosis in HIV-infected individuals. Its diagnostic effectiveness can be improved by modifying and optimizing the assay design.

## Introduction

Human immunodeficiency virus (HIV) infection causes significant damage to the immune function of patients, especially destroying T cells, leading to a significant increase in the incidence of various diseases [1]. Tuberculosis is a chronic infectious disease with the highest morbidity and mortality in China, which has caused 1.7 million deaths annually [2]. As a result of resistant deficiency, HIV-infected patients are highly susceptible to tuberculosis. These two diseases interact and promote each other, exacerbating the overall level of symptoms [3]. World Health Organization (WHO) statistics indicate that about 30% of HIV patients are simultaneously infected with Mycobacterium tuberculosis (Mtb).

The interferon-*γ* release assay (IGRA) is a method that detects interferon *γ* (IFN-*γ*) released from specific T cells after peripheral blood mononuclear cells are stimulated by tuberculosis-specific antigens. Because of its high sensitivity and high specificity [4, 5], IGRA has been applied in the clinical diagnosis of tuberculosis infection, and introduced by many guidelines and recommendations. The clinical atypia of HIV-infected individuals complicated with tuberculosis increases the difficulty in diagnosing tuberculosis, and therefore IGRA becomes an essential auxiliary examination in the diagnosis. Two types of IGRA, QuantiFERON-TB Gold In-Tube (QFT-GIT) approved by FDA of USA and T-SPOT.TB used in Europe, are currently used for the detection of tuberculosis [6, 7]. These two assays are based on the early secretory antigenic target 6 (ESAT-6) secreted by Mtb, and the good immunogenicity of culture filtrate protein-10 (CFP-10). ESAT-6 and CFP-10 are endemic to Mtb, but absent in Bacillus Calmette-Guerin (BCG) and most environmental mycobacteria. Additionally, they can induce the secretion of IFN-*γ* by lymphocytes in tuberculosis patients. Then, these T cells producing IFN-*γ* can be detected by sensitive enzyme-linked immunosorbent assay (ELISA) or ELISPOT.

In 2016, Huo *et al*. showed that the IGRA did not appear to be optimal for the clinical confirmation of active tuberculosis infection in HIV-infected individuals [8]. Among the two types of IGRA, the T-SPOT.TB assay seems to be more accurate in distinguishing active tuberculosis infection in HIV-infected individuals, while the QFT-GIT assay reduces indeterminate results. There are still numerous related high-quality studies published after 2016.

By using meta-analysis, this study systematically evaluated the published trials on IGRA for diagnosing tuberculosis infection in HIV-infected patients, thus objectively assessing the clinical diagnostic value of IGRA, and consequently providing a reference for its clinical application.

## Methods

### Search strategy

This meta-analysis was carried out in accordance with the preferred reporting items for systematic reviews and meta-analyses (PRISMA) [9]. Relevant studies on IGRA for diagnosing tuberculosis in HIV-infected patients were comprehensively searched and collected from Excerpta Medica Database (EMBASE), Medline, Cochrane Library, Chinese Sci-tech Periodical Full-text Database, Chinese Periodical Full-text Database, China National Knowledge Infrastructure (CNKI) and China Wanfang Data up to July 2020. The following keywords were utilised: ‘tuberculosis’, ‘interferon-gamma release assay’, ‘QFT-GIT’, ‘T-SPOT.TB’, ‘IGRA’, ‘HIV’, ‘acquired immune deficiency syndrome’. Additional references could be collected from reviews, guides and conferences. The comprehensive database search was completed independently by the two researchers (YYM and YNX). There was no language limit.

### Inclusion and exclusion criteria

#### Inclusion criteria

1. Evaluation of IGRA in the diagnosis of HIV-associated active tuberculosis infection;
2. Application of QFT-GIT or T-SPOT.TB;
3. More than 10 HIV-associated active tuberculosis infection patients;

#### Exclusion criteria

1. The survey did not establish a control group among HIV-seropositive subjects;
2. No original data included in a study;
3. Prospective research reports, conferences summaries, reviews, guidelines and case reports.

### Literature quality assessment

The quality of all included studies was assessed using the Quality Assessment of Diagnostic Accuracy Studies-2 (QUADAS-2) tool, a standard widely used tool for clinical trials of diagnostic accuracy [10]. Specifically, each item is rated as ‘yes’, ‘no’ or ‘unclear’; ‘yes’ indicates that the item satisfies quality criterion, ‘no’ suggests no satisfaction or no related information mentioned, and ‘unclear’ is defined as partial satisfaction or insufficient information from the literature.

### Data extraction

The two researchers (XQC and XJC) independently extracted all the data, and then cross-checked. During this process, disagreement was resolved through discussion between the two. The following information was extracted: author, year of publication, country, language, age group of subjects, IGRA type. Additionally, a table was designed to extract sensitivity, specificity, true-positive number, false-positive number, false-negative number, true-negative number.

### Statistical analysis

Statistical analysis was completed by using Stata 15.0 statistical software. Heterogeneity among studies was evaluated by the inconsistency index (*I*^2^) and its *P*-value. Effects of diagnostic threshold were assessed by the summary receiver operating characteristic (SROC) curve and Spearman's correlation coefficient between the logarithm of sensitivity and logarithm of 1-specificity. The pooled sensitivity, specificity, positive likelihood ratio (PLR), negative likelihood ratio (NLR) and diagnostic odds ratio (DOR) were calculated using a bivariate mixed-effects model, and the corresponding forest plots were developed. The area under the curve (AUC) was obtained. Finally, Deeks' funnel plot was used to assess publication bias.

## Results

### Literature research, characteristic and quality of studies

**Fig. 1.**
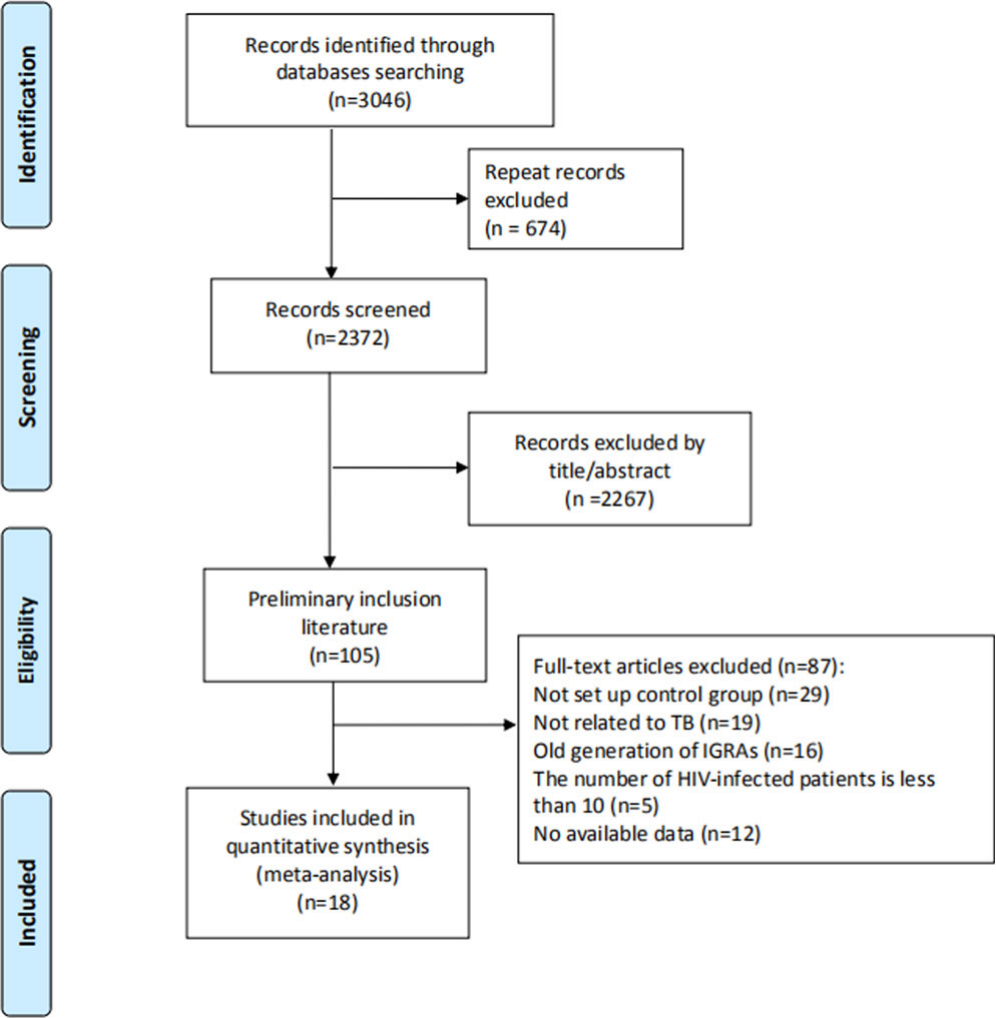
A flow chart of the literature selection process.

**Fig. 2.**
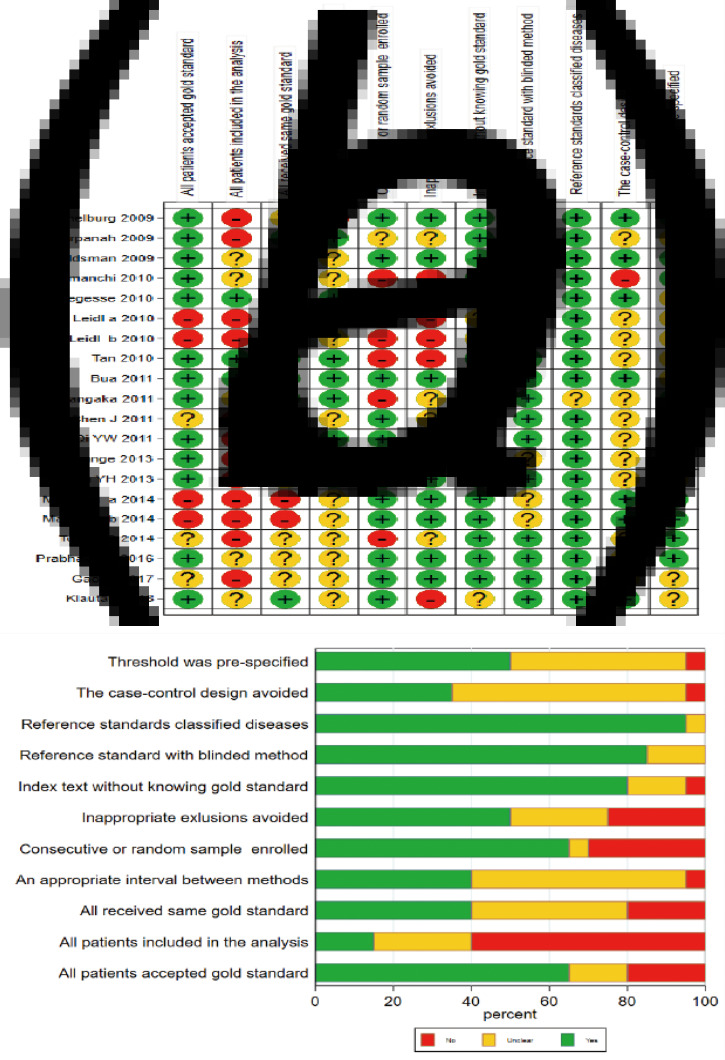
Methodological quality evaluation results of the included studies using the Quality Assessment of Diagnostic Accuracy Studies-2 (QUADAS-2) tool. (A: Risk of bias summary; B: Risk of bias graph).

Initially, 3046 articles were retrieved by keywords, and then 674 duplicate articles were excluded. Next, 2267 articles were excluded by reading titles and abstracts, and 87 articles by reading full text and checking data integrity. Finally, 18 articles including 20 trials [11–28] were included in this meta-analysis, with QFT-GIT in 11 trials and T-SPOT.TB in nine trials. The specific screening flowchart is shown in Figure 1. The basic characteristics of the included literature are shown in Table 1. The quality assessment result is shown in Figure 2. There were nine studies that showed a higher risk of bias, mainly concentrated in the item: consecutive or random sample of patients enrolled and all patients included in the analysis. More detailed information could be found in Figure 2.

**Table 1.** General characteristics of the reviewed studies and the primary results

Study	Year	Country	ATB/non-ATB patients enrolled	Age(years)		CD4+ counts (cells/μL3)			Assay results						
				ATB patients	Non-ATB patients	ATB patients	Non-ATB patients	IGRA method	Sensitivity	Specificity	TP	FP	FN	TN	I
Aichelburg [11]	2009	Austria	8/822	39 (32–47)	39 (32–47)	393 (264–566)	393 (264–566)	QFT-IT	0.88	0.95	7	37	1	738	47
Davarpanah [12]	2009	Iran	11/165	38 (23–60)	38 (23–60)	360 (34–1300)	360 (34–1300)	QFT-IT	0.36	0.70	4	45	7	107	13
Veldsman [13]	2009	South Africa	30/30	NR	NR	NR	NR	QFT-IT	0.38	0.59	9	11	15	16	9
Cattamanchi [14]	2010	Uganda	112/100	33 (27–40)	33 (27–40)	49 (16–160)	49 (16–160)	T-SPOT	0.73	0.54	61	34	23	40	54
Legesse [15]	2010	Ethiopia	6/21	34.2 (18–70)	34.2 (18–70)	NR	NR	QFT-IT	0.83	0.50	5	10	1	10	1
Leidl a [16]	2010	Uganda	19/109	33.4 ± 6	34.1 ± 8.3	182 (118)	283 (226)	T-SPOT	1.00	0.44	17	59	0	46	6
Leidl b [16]	2010	Uganda	19/109	33.4 ± 6	34.1 ± 8.3	182 (118)	283 (226)	QFT-IT	0.68	0.30	13	74	6	31	4
Tan [17]	2010	China	9/30	39.6 (30–52)	39.1 (19–67)	112 (31–332)	145 (0–596)	T-SPOT	0.89	0.93	8	2	1	28	0
Chen J [18]	2011	China	38/109	41 (35–53)	39 (31–47)	35 (14–83)	92 (27–163)	T-SPOT	0.37	0.89	13	11	22	86	15
Bua [19]	2011	Italy	9/64	41 (21–63)	41 (21–63)	270 (4–897)	270 (4–897)	QFT-IT	0.25	0.89	2	6	6	47	12
Rangaka [20]	2011	South Africa	50/729	35 (31–40)	36 (31–42)	169 (98–239)	198 (136–315)	QFT-IT	0.68	0.56	32	298	15	383	51
Qi YW [21]	2011	China	40/54	34.4 ± 9.2	34.4 ± 9.2	NR	NR	T-SPOT	0.58	0.24	23	41	17	13	0
Yu YH [22]	2013	China	34/42	40.2 ± 7.5	40.7 ± 15.4	92.5 ± 130.9	134.7 ± 146.3	T-SPOT	0.38	0.93	13	3	21	39	0
Lagrange [23]	2013	India	38/54	38.0 (30.5–42.0)	38.0 (30.5–42.0)	NR	NR	QFT-IT	0.90	0.60	26	19	3	29	15
Markova a [24]	2014	Bulgaria	13/52	43 (29–63)	37 (21–66)	195 (15–450)	248 (36–785)	T-SPOT	0.89	1.00	8	0	1	48	8
Markova b [24]	2014	Bulgaria	13/52	43 (29–63)	37 (21–66)	195 (15–450)	248 (36–785)	QFT-IT	0.92	1.00	12	0	1	49	3
Tong XC [28]	2014	China	45/30	45 ± 12	47 ± 15	NR	NR	T-SPOT	0.80	0.77	36	7	9	23	0
Prabhavathi [25]	2016	India	55/53	21–52	19–56	NR	NR	QFT-IT	0.73	0.46	30	25	11	21	21
Gao M [26]	2017	China	55/82	NR	NR	NR	NR	T-SPOT	0.93	0.99	25	1	2	109	0
Klautau [27]	2018	Brazil	80/10	39.2 ± 10.9	39.2 ± 10.9	557.5 ± 283.89	557.5 ± 283.89	QFT-IT	0.94	1.00	75	0	5	10	0

IGRA, *γ*-interferon release assay; TP, true-positive; FP, false-positive; FN, false-negative; TN, true-negative; NR, not reported; QFT-GIT, Quantiferon-TB Gold In-Tube; ATB, active tuberculosis; I, indetermination.

### Meta-analysis

**Fig. 3.**
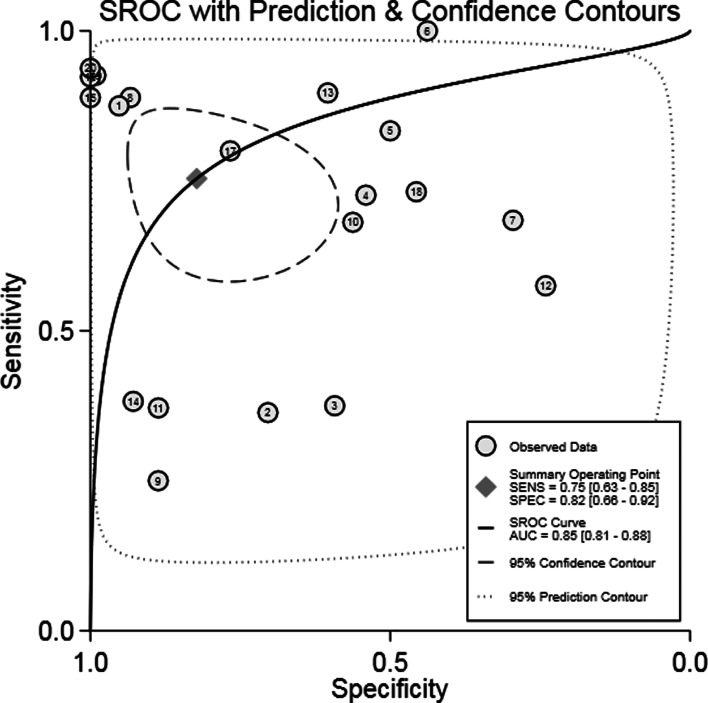
SROC curve for the accuracy of IGRA in the diagnosis of tuberculosis in HIV-seropositive individuals. SROC, summary receiver operating characteristic; IGRA, interferon-*γ* release assay; HIV, human immunodeficiency virus.

**Fig. 4.**
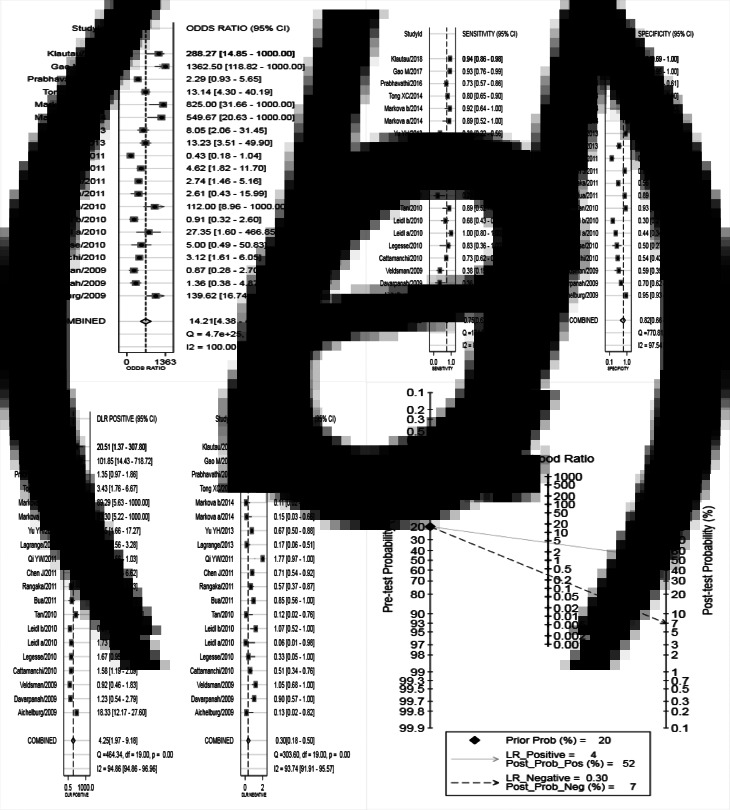
Forest plots of IGRA in the diagnosis of tuberculosis in HIV-seropositive individuals (DOR (a), sensitivity and specificity (b), PLR and NLR (c), Fagan's Nomogram (d) of IGRA for diagnosing active tuberculosis in HIV-seropositive individuals). IGRA, interferon-*γ* release assay; HIV, human immunodeficiency virus; DOR, diagnostic odds ratio; PLR, positive likelihood ratio; NLR, negative likelihood ratio.

Analysis of diagnostic accuracy showed significant heterogeneity in sensitivity (*P* = 0.00, *I*^2^ = 89.14%), specificity (*P* = 0.00, *I*^2^ = 97.54%), PLR (*P* = 0.00, *I*^2^ = 94.86%), NLR (*P* = 0.00, *I*^2^ = 93.74%) and DOR (*P* = 0.00, *I*^2^ = 100.0%). No significant threshold effect was found in the current meta-analysis, because the SROC curve was not a typical ‘shoulder arm’ pattern (Fig. 3), and Spearman correlation coefficient of the logarithm of sensitivity and logarithm of 1-specificity was 0.312 (*P* = 0.181). Overall, the diagnostic accuracy of IGRA for HIV-associated active tuberculosis was as follows: pooled sensitivity = 0.75 (95% CI 0.63–0.85), pooled specificity = 0.82 (95% CI 0.66–0.92), PLR = 4.25 (95% CI 1.97–9.18), NLR = 0.30 (95% CI 0.18–0.50), DOR = 14.21 (95% CI 4.38–46.09). The forest plots of DOR, sensitivity and specificity, PLR and NLR are shown in Figure 4a–c, respectively.

**Fig. 5.**
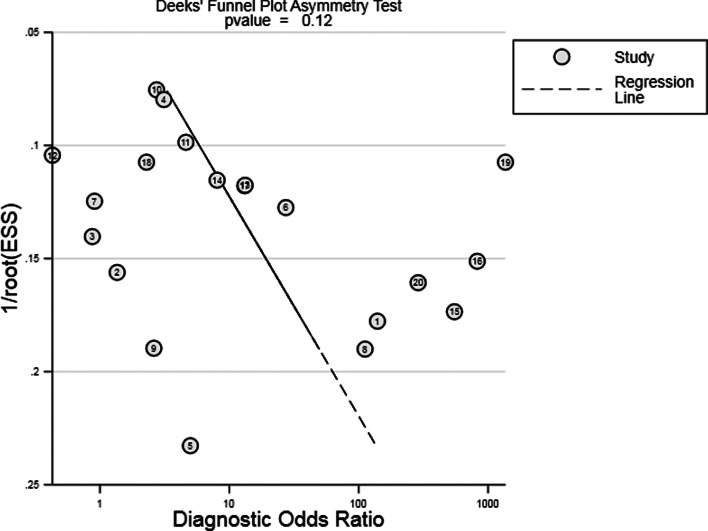
The Deeks' funnel plot asymmetry test for the evaluation of publication bias.

The SROC curve was shown in Figure 3, and the AUC was 0.85. Fagan's Nomogram result indicated that, when the current test probability was 20%, the post-test possibility of PLR was 52% while that of NLR was 7% (Fig. 4d). This result suggested that IGRA had a specific diagnostic performance for HIV complicated with tuberculosis infection. Deeks funnel plot in Figure 5 suggested low publication bias (*P* > 0.05).

### Meta-regression and subgroup analysis

**Fig. 6.**
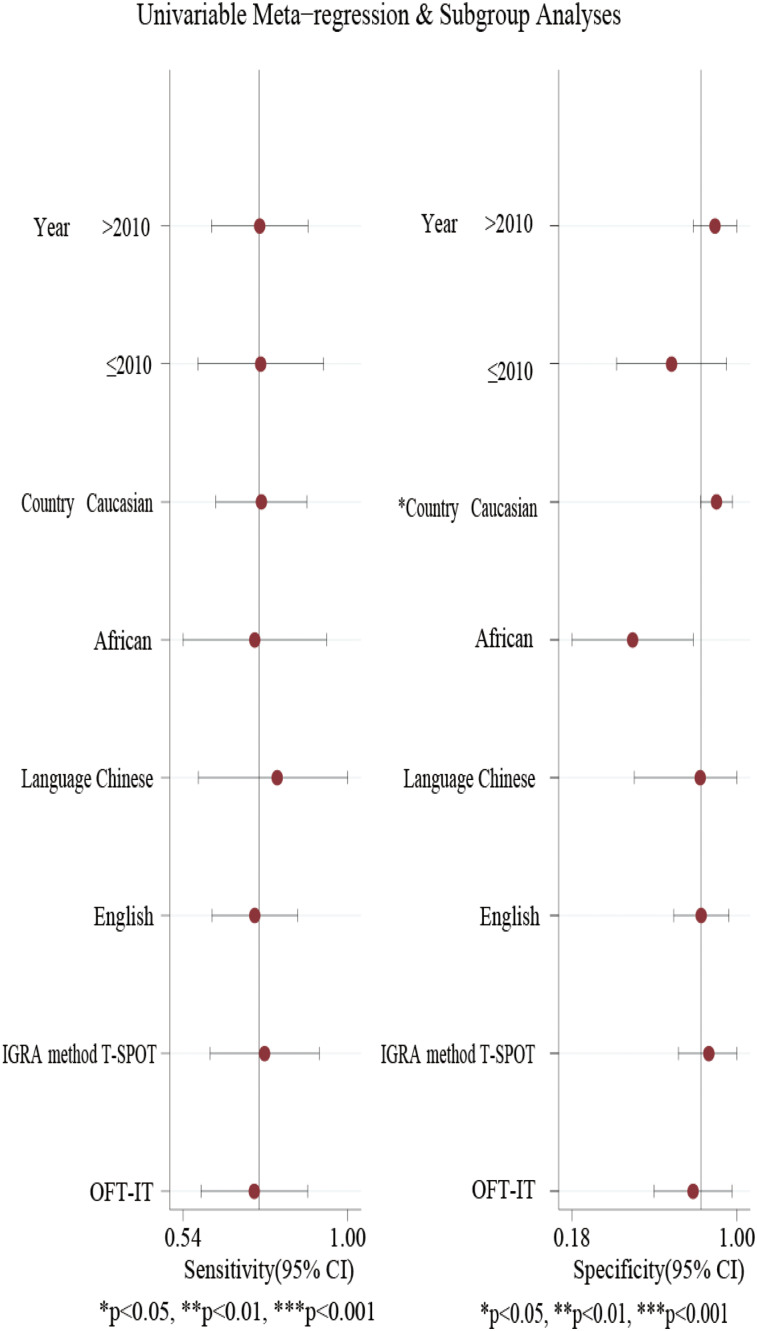
Meta-regression and subgroup analyses.

Further, meta-regression and subgroup analysis were carried out to explore the source of heterogeneity. As shown in Figure 6, Caucasian, Asian and African had *P*-value <0.05 in the pooled specificity, suggesting significant heterogeneity among ethnic groups. No statistical difference was identified in the other three subgroups (publication year, language and IGRA method). Meta-regression analysis showed that only the variable ethnicity was statistically significant. Collectively, ethnicity might be the source of the heterogeneity.

## Discussion

Tuberculosis is the most common opportunistic infection in HIV-infected patients. It is the leading cause of death, especially in developing countries where the HIV epidemic has led to a rise in tuberculosis prevalence. The atypical clinical symptoms of HIV-infected patients with tuberculosis result in high incidence of extrapulmonary tuberculosis and co-infection with other pathogens, and difficulty in obtaining etiological evidence. The effective way to control tuberculosis is to find a quick and simple diagnostic method with high sensitivity and high specificity. In the past, the PPD test and tuberculosis antibody test were used to assist tuberculosis diagnosis. However, the PPD test has certain limitations. Some components of it have common antigens with BCG and most non-tuberculous mycobacteria, so the diagnostic specificity of the PPD test is relatively low. Additionally, as a result of immunodeficiency in HIV patients, the sensitivity of the PPD test is also insufficient, and the false-negative rate is high [29]. Moreover, the operation of PPD test is affected by human factors and takes a long time (48–72 h). Collectively, PPD test is not an optimal diagnostic method for tuberculosis. Similarly, a recent study [30] proved that the sensitivity and specificity of the tuberculosis antibody test, a humoral immunoassay, are not high, and therefore its diagnostic value is limited. By contrast, IGRA is a new T-cell-based immunological test developed in recent years. Specific antigens of Mtb used in the IGRA test are an antigenic polypeptide that encodes the RD1 gene of Mtb. The binding RD1 genes in BCG, as well as in the vast majority of non-tuberculosis branches, are missing in the genome, which could theoretically avoid that TST cross-antigen reaction affects specificity in tuberculin test. IGRA can distinguish between real tuberculosis infection and BCG vaccination-induced [31]. Luo *et al*. [32] declared that the diagnostic sensitivity of T-SPOT.TB was 92.3% and the specificity was 85.6%, indicating that IGRA had a certain diagnostic value in tuberculosis. Currently, T-SPOT.TB has been used in many countries for auxiliary diagnosis of active tuberculosis and has been applied in a number of hospitals in China. IGRA can provide additional clinical evidence for the auxiliary diagnosis of tuberculosis infection in HIV-infected patients.

This comprehensive analysis selected a total of 18 articles including 20 trials (652 HIV-infected patients with suspected tuberculosis, 2566 controls). Specifically, the pooled sensitivity of IGRA in diagnosing active tuberculosis infection in HIV patients was 0.75, indicating that the rate of missed diagnosis and misdiagnosis was 25%. The pooled specificity was 0.82, suggesting that nearly 20% of patients without tuberculosis infection may be diagnosed with tuberculosis infection. The pooled PLR was 4.25, indicating that IGRA correctly judged positive 4.25 times more likely than wrong judgment. The pooled NLR was 0.30, indicating that the probability of wrong judgment of negative was 30% of correct assessment. The +LR more than 10, and the –LR less than 0.1 showed convincing accuracy [33], indicating that serum IGRA is of limited value in diagnosing active tuberculosis infection in HIV patients. The value of DOR ranges from 0 to infinity, and a higher value means better disease diagnostic potential [34]; DOR of IGRA was 14.21, suggesting that IGRA could be a method for diagnosing tuberculosis infection in HIV patients. AUC is an index to evaluate the overall effectiveness of diagnostic tests, and an AUC value between 0.93 and 0.96 indicates an excellent diagnostic capability [35, 36]; in this study, the area under SROC curve was 0.85, suggesting that the diagnostic efficacy is limited, which is similar to the results of the study in 2016 [8]. Based on this previous meta-analysis, our study adopted more strict literature inclusion standards, and included new studies published after 2016. Moreover, our study eliminated the publication bias by increasing the numbers of papers.

The Spearman correlation coefficient suggested that the heterogeneity was not caused by the threshold effect. The meta-regression and subgroup analysis indicated that ethnicity was the source of heterogeneity in this study. High pooled specificity was found in Caucasian and Asian. However, the publication year, language and IGRA method had no effects on the results.

However, there are still some limitations in this study: (1) The number of included literature is small, which may not summarise the research progress in this field well. (2) The included literatures are all published research, while high-quality unpublished researches are not included and analysed, which may lead to publication bias. (3) Heterogeneity exists in this study, which may affect the diagnosis accuracy of IGRA in HIV-associated tuberculosis infection. (4) A considerable number of participants in the included articles had indeterminate IGRA results, which might affect the diagnosis accuracy of IGRA in patients with HIV-associated tuberculosis infection.

In conclusion, IGRA has a good diagnostic value and therefore can aid in preliminary screening of active tuberculosis in HIV-infected individuals. Due to the limitations of this study, the above results need to be further confirmed by more well-designed and scientific clinical trials.

## Data Availability

The datasets used and/or analysed during the current study are available from the corresponding author on reasonable request.
